# The Influence of College Students' Empathy on Prosocial Behavior in the COVID-19 Pandemic: The Mediating Role of Social Responsibility

**DOI:** 10.3389/fpsyt.2021.782246

**Published:** 2021-12-02

**Authors:** Yanfei Jiang, Youjuan Yao, Xiaoliang Zhu, Shiling Wang

**Affiliations:** ^1^Key Laboratory of Behavior and Mental Health of Gansu Province, Department of Psychology, Northwest Normal University, Lanzhou, China; ^2^Department of Psychology, Northwest Normal University, Lanzhou, China

**Keywords:** prosocial behavior, empathy, social responsibility, COVID-19, mental health

## Abstract

Prosocial behavior has played an irreplaceable role during the COVID-19 pandemic, not only in infection prevention and control, but also in improving individual mental health. The current study was conducted after COVID-19 control was under the stage of Ongoing Prevention and Control in China. Using the Interpersonal Response Scale, Prosocial Tendencies Measure and Big Five Personality Questionnaire. In total, 898 college students participated in the current study (*M*age = 19.50, *SD*age = 1.05, Age range = 16–24). The result showed that against the background of the COVID-19 pandemic, college students' social responsibility partially mediated the relationship between empathy and prosocial behavior. This study provides new insights and inspiration for improving college students' mental health in the context of the pandemic.

## Introduction

The global outbreak of COVID-19 began in December 2019 (1). The World Health Organization has classified the COVID-19 outbreak as a Public Health Emergency of International Concern (2). COVID-19 triggered a psychological crisis on an unprecedented global scale, especially for college students who faced many challenges (3, 4). Due to the negative impact of the pandemic and the subsequent lockdowns, college students have experienced a transition from physical classes to online remote classes, the loss of daily social activities, and the greater pressure of employment, which have significantly affected their mental health, normal interpersonal activities and social life (3–5). Thus, against the background of the COVID-19 pandemic, it is important to investigate the factors that improve college students' mental health.

When individuals are threatened by natural disasters or health crises, prosocial behavior can be a positive factor in improving individual mental health. Prosocial behavior can promote individual life satisfaction, happiness, mental health, and other psychological states (6). Meanwhile, prosocial behavior interventions can promote individual mental health (7), and reduce individual depression and anxiety levels (6). Furthermore, high level of prosocial behavior has positive effects on both helpers and recipients (8), not only providing benefits to the recipient, but also boosting the givers happiness and health, thus helping to cope with the deadly coronavirus (9). Therefore, against the background of the COVID-19 pandemic, promoting college students prosocial behaviors is a viable way to maintain mental health, which is of great significance. However, little is known about the prosocial behavior of college students in the context of COVID-19 (8). Therefore, exploring prosocial behavior and its influencing factors among college students in the context of COVID-19 plays a significant role in promoting college students' mental health and disease prevention and control.

Quantitative studies have shown that empathy is closely related to prosocial behavior. Empathy ability can positively predict individual' s prosocial behavior (10). Highly empathetic individuals exhibited more prosocial behaviors (11–13). They are more attentive to the feelings and needs of others (14). In order to avoid feelings of guilt over unhelpful thoughts and actions, individuals may exhibit more prosocial behaviors. Furthermore, empathy is the common motivational basis of prosocial behavior (15). For example, during the COVID-19 pandemic, empathy romotes the motivation of individuals' prosocial behavior of wearing a face mask and maintaining physical distance (16, 17).

Empathy may promote prosocial behavior through a specific pathway. It is found that level of empathy positively predicts the degree of social responsibility (18, 19). There was is a moderate positive correlation between empathy and responsibility. That is, individuals with higher level of empathy have higher level responsibility (20). Furthermore, Chapman et al. (21) showed that the perception of another' s pain and the responsibility to the person in need might trigger prosocial behavior. Social responsibility acts as an important influence on individual helping behaviors (22), it is activated by situational and individual factors, and the level of activation determines the level of prosocial behavior (23). In addition, responsibility is an effective predictor of a series of positive psychology and behaviors such as altruism (24, 25). Individuals with higher social responsibility have higher level of prosocial behavior (23). We hypothesized that social responsibility may explain the relationship between empathy and prosocial behavior.

Based on the literature review, we proposed the following hypotheses:

Hypothesis 1. In the context of the COVID-19 pandemic, there is a significant positive correlation between empathy, social responsibility, and prosocial behavior among college students.
Hypothesis 2. In the context of the COVID-19 pandemic, social responsibility plays a mediating role in the effect of empathy on prosocial behavior.

## Materials and Methods

### Participants

Fighting COVID-19 China in Action indicates that since April 29, 2020, COVID-19 control has been conducted on the ongoing prevention and control in China (26). The current study was conducted after the pandemic was basically controlled and normal daily life was restored in China. We Investigated the empathy, social responsibility and prosocial behavior of college students from September 2020 to March 2021. Data were collected by Questionnaire Star platform and offline paper questionnaire. In total, 898 (*M*_age_ = 19.50, *SD* = 1.05, Range = 16–24 years, 66.4% female) college students from Northwest Normal University completed the test anonymously. All participants in the current study were informed consent.

### Measures

#### Empathy

Empathy was measured by the Interpersonal Reactivity Index-C (IRI-C), designed by Davis (27) and revised by Zhang Fengfeng et al. (28). The scale has 22 items. In total, rated on a five-point Likert scale ranging from one (complete disagreement) to five (complete agreement). A higher score indicates a higher level of empathy. This questionnaire includes four dimensions: viewpoint selection, empathic fantasy, empathic concern, and personal pain. Cognitive empathy is measured by viewpoint selection and empathic fantasy, and emotional empathy is measured by empathic concern and personal pain. This scale has been proven to have good reliability and validity in previous studies (28). The Cronbach α coefficients of this questionnaire in the current study was 0.76, the Cronbach α coefficients of cognitive empathy was 0.7, and the Cronbach α coefficients of emotional empathy was 0.61. Confirmatory factor analysis (CFA) suggested that the corrected model fit the data well: χ^2^ = 382.917, χ^2^*/df* = 3.868, CFI = 0.952, NFI = 0.937, RFI = 0.854, IFI = 0.953, RMSEA = 0.057.

#### Prosocial Behavior

Prosocial behavior was measured using the Prosocial Tendencies Measure (PTM) designed by Carlo (29) and revised by Cong Wenjun (30). The questionnaire has 23 items, rated on a five-point Likert scale ranging from one (complete disagreement) to five (complete agreement). The questionnaire had six dimensions: anonymity, altruism, openness, compliance, urgency, and emotional prosocial behavior. A higher score indicates a higher frequency of prosocial behavior. The scale has been proven to have good reliability and validity in practice (30). The Cronbach α coefficient of this questionnaire in the current study was 0.84. Confirmatory factor analysis (CFA) suggested that the corrected model fit the data well: χ^2^= 845.262, χ^2^*/df* = 4.449, CFI = 0.883, NFI = 0.855, RFI = 0.808, IFI = 0.884, RMSEA = 0.062.

#### Social Responsibility

Social responsibility was measured by the “conscientiousness” subscale of John's Big Five Inventory (BFI). The scale consists of 12 items, rated on a five-point Likert scale ranging from one (complete disagreement) to five (complete agreement), with higher scores indicating higher levels of social responsibility. The scale has good reliability and validity (31, 32), The Cronbach α coefficient of the subscale in the current study was 0.69. Confirmatory factor analysis (CFA) suggested that the corrected model fit the data well: χ^2^ = 155.053, χ^2^*/df* = 3.524, CFI = 0.970, NFI = 0.959, RFI = 0.938, IFI = 0.970, RMSEA = 0.053.

### Data Analysis

Data were analyzed using SPSS 24.0. Since empathy, social responsibility, and prosocial behavior were all measured by self-reported scales, there was a possibility that this may lead to common method bias effects (33). Therefore, the current study used anonymous measurements and reverse-scoring to control from program. After data collection, the Harman univariate test was used to test the size of the common method deviation. Unrotated exploratory factor analysis results extracted a total of 12 factors having eigenvalue roots greater than one, and the maximum factor variance explanation rate was 16.76%, lower than the critical standard of 40%, indicating that there was no obvious common method bias in the current study. Next, descriptive statistics and correlation analyses were performed for the data in the current study. On this basis, the macro program Process 3.4 was used to test the mediating effect of social responsibility on empathy and prosocial behavior.

## Results

### Preliminary Analysis

Independent samples *t*-test was used to test for gender differences. The results showed that there were significant gender differences in empathy, cognitive empathy, and emotional empathy. Female students' scores on empathy (*t* = −4.60, *p* < 0.001), cognitive empathy (*t* = −3.18, *p* < 0.05) and emotional empathy (*t* = −4.75, *p* < 0.001) were significantly higher than those of males. But there were no significant gender differences in social responsibility (*t* = −0.91, *p* = 0.363) and prosocial behavior (*t* = 1.62, *p* = 0.105).

One-sample *t*-test was used to investigate the differences of the empathy, prosocial behavior and social responsibility between our study and the previous studies. The mean score of empathy of college students in this study (3.28 ± 0.47) was lower than that of Huang S et al. (18) (3.35 ± 0.37), which was statistically significant (*t* = −4.57, *p* < 0.001). The mean score of prosocial behavior (3.14 ± 0.51) was lower than that of Li L et al. (34) (3.14 ± 0.51), which was statistically significant (*t* = 0.02, *p* < 0.001). However, the mean score of social responsibility (3.43 ± 0.47) was higher than the national norm of responsibility (3.35 ± 0.56) (32), which was statistically significance (*t* = 5.02, *p* < 0.001).

### Correlation Analysis of Empathy, Prosocial Behavior and Social Responsibility

Table 1 shows the means, standard deviations, and Pearson correlation coefficients for all variables in the current study. Correlation analysis showed that, in the COVID-19 pandemic, there was a significant positive correlation between empathy and prosocial behavior, empathy and social responsibility, and social responsibility and prosocial behavior of college students, which confirmed Hypothesis 1.

**Table 1:** Descriptive statistics and Pearson correlations for all measures.

**Measure**	***M***	***SD***	**1**	**2**	**3**
1 Empathy	3.28	0.47	1		
2 Prosocial behavior	3.14	0.51	0.389^**^	1	
3 Social responsibility	3.43	0.47	0.168^**^	0.320^**^	1

*p <0.05,

** p <0.01, ***p <0.001.

### The Mediating Effect of Social Responsibility

The results of the correlation analysis showed that there were significant correlations among empathy, social responsibility, and prosocial behavior of college students in the current study, which met the conditions of the mediation effect analysis. Next, Model 4 in SPSS macro prepared by Hayes was used to conduct a mediation analysis with gender and grade as covariates, social responsibility as a mediating variable, empathy as an independent variable, and prosocial behavior as a dependent variable. The results are shown in Table 2. Empathy positively predicts social responsibility and prosocial behavior, while social responsibility positively predicts prosocial behavior.

**Table 2:** The results of regression analysis of variables in this study.

**Predictors**	**Model 1 (SR)**		**Model 2 (PB)**		**Model 3 (PB)**	
	**β (95%CI)**	***t***	**β (95%CI)**	***t***	**β (95%CI)**	***t***
Gender	−0.039(−0.106,0.028)	−1.147	−0.103(−0.168,0.037)	−3.091^*^	−0.113(−0.181,−0.046)	−3.291^*^
Grade	0.027(0,0.041)	1.957	0.010(−0.017,0.036)	0.727	0.017(−0.010,0.045)	1.237
EP	0.178(0.113,0.243)	5.389^***^	0.391(0.327,0.456)	11.973^***^	0.441(0.375,0.506)	13.182^***^
SR			0.276(0.212,0.340)	8.493^***^		
*R^2^*	0.036		0.228		0.166	
*F*	11.041^***^		65.973^***^		59.208^***^	

*N, 449; SR, social responsibility; PB, prosocial behavior; EP, empathy; Gender was dummy coded such that 0, female; 1, male; Grade was dummy coded such that 0, first year; 1, second year; 2, third year; 3, fourth year; 4, last year; 5, postgraduate*.

* *p <0.05,*

**p <0.01,

*** *p <0.001*.

**Figure 1:**
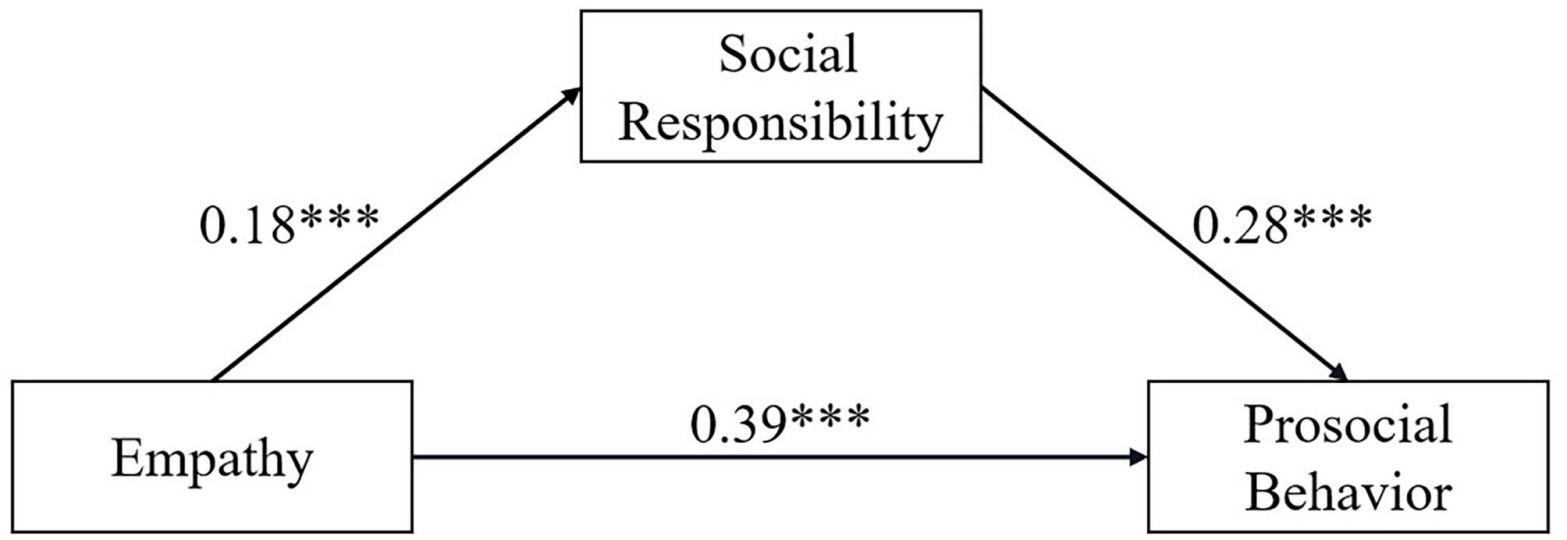
Mediation effect model of social responsibility between empathy and prosocial behavior.

The bootstrap method was used to test the mediating effects of the data collected in this study. The sample size was 5,000. Under the 95% confidence interval, the total effect of empathy on prosocial behavior was 0.4406. The direct effect result did not contain 0 (LLCI = 0.3227, ULCI = 0.4582), indicating that the direct effect was significant, and the direct effect size was 0.3914. The results of the mediating effect did not contain 0 (LLCI = 0.0281, ULCI = 0.0731), indicating that the mediating effect of social responsibility was significant. The size of the mediating effect was 0.0492, accounting for 11.2% of the total effect of empathy on prosocial behavior, as shown in Table 3. Social responsibility plays a partially mediating role in the relationship between empathy and prosocial behavior, and Hypothesis 2 of this study was confirmed. As shown in Figure 1, empathy can directly predict prosocial behavior, and social responsibility enhances the predictive effect of empathy on prosocial behavior.

**Table 3:** Testing the mediating role of social responsibility.

**Effect type**	**Effect size**	***Boot SE***	**Bootstrap 95% CI**		**Proportion of effect size**
			***Boot LLCI***	***Boot ULCI***	
Total effect	0.4406	0.0375	0.3684	0.5142	
Direct effect	0.3914	0.0344	0.3227	0.4582	88.83%
Indirect effect	0.0492	0.116	0.0281	0.0731	11.17%

## Discussion

The current study aimed to examine the relationship between empathy and prosocial behavior and the mediating role of social responsibility among college students in the context of COVID-19. The results showed that college students' social responsibility partially mediated the relationship between empathy and prosocial behavior. The result is helpful to popularize the cultivation of prosocial behavior among college students in the context of COVID-19 and its positive significance for mental health and pandemic prevention and control.

The study found that female students scored significantly higher in empathy, cognitive empathy and emotional empathy than male students, which is consistent with previous studies (35, 36). Gender differences in empathy may be related to the defects of self-reported scale. Male participants answer items with feminine characteristics on the IRI-C (softheartedness, worry, and fear) less honestly because they are unwilling to admit that they have “feminine” thoughts, feelings, or behaviors (36). At the same time, the study showed that in the context of the COVID-19 pandemic, college students' empathy is lower than it was before its outbreak (18), which may be linked to the excessive internet use caused by the policy of long-term at-home quarantine during the pandemic. Because excessive internet use had negative effects on empathy (37). In addition, the long-term home quarantine policy also blocks normal interpersonal communication among college students, and the loss of face-to-face contact for a long time may lead to the decline of individual social sensitivity and thus impair individual empathy (38).

Social responsibility of college students in the current study is higher than the norm level. This is consistent with previous studies in the context of COVID-19 pandemic (39, 40), college students have a high level of social responsibility in the context of pandemic, which may be related to the government' s education on social responsibility when there are major public health emergencies.

In this study, the prosocial behavior of college students in the context of pandemic is lower than that of college students before the pandemic (34). This may be related to the maladaptation of college students in the context of the pandemic. Students with better school adaptability had more prosocial behaviors (41), while maladaptation will reduce the probability of the occurrence of prosocial behaviors. In the context of the pandemic, Chinese college students have experienced the transition from online classes to physics classes. This results in maladjustment of college students and negative influence on prosocial behavior. In addition, the novel coronavirus human-to-human transmission characteristics (42) require colleges students to maintain a set mandatory physical distance from each other, which may lead to a decrease in the frequency of prosocial behavior.

The study results showed that empathy levels of college students in the context of pandemic can significantly positively predict prosocial behavior, and the higher the level of empathy, the more prosocial behavior, which is consistent with previous research results on college students' prosocial behavior (43, 44). According to the Empathy-Altruism hypothesis, when an individual empathizes with others, they will experience events and emotions by stepping into people's shoes, thus arousing the pure altruistic motivation of the individual and encouraging the individual to help others regardless of the cost (45). This suggests that empathy is an important motivational basis for prosocial behavior (15, 16). Meanwhile, some researchers believe that individuals engage in prosocial behaviors to alleviate intrapsychic pain caused by empathy (13). However, no matter what kind of the motivation is, empathy has a positive impact on prosocial behavior, which then promotes individual mental health (6, 46). Therefore, in the context of COVID-19, cultivation of student empathy levels effectively promotes students' prosocial behaviors.

The results showed that social responsibility plays a partial mediating role in the effect of empathy on prosocial behavior. Under the background of pandemic, college students' empathy ability can enhance the expression effect of social responsibility, thus increasing the frequency of prosocial behavior. The anterior radius of the mediation model showed that empathy can positively predict social responsibility, which is consistent with the results of previous studies (18, 47). In the context of the pandemic, college students directly or indirectly feeled the disaster and pain brought by novel Coronavirus to others, and their empathy for the victims inspires their high sense of social responsibility. The posterior radius of the mediation model showed that social responsibility can positively predict prosocial behavior, which is consistent with previous study (23). This may be because individuals who feel the pain of the victim and have the responsibility to the person in need engage in more prosocial behaviors (22). College students are in a period when their values are forming and becoming stable. Role models, social conditions and cultural background have an important influence on the formation of their faiths (48). In the context of the pandemic, scientific research workers, paramedics, firefighters, and other groups with a high degree of social responsibility could be appropriate examples of social responsibility for college students. Follow such workers could produce more prosocial behaviors in college students, which would in turn contribute to the prevention and control of the pandemic and support for college students' mental health.

The results showed that social responsibility plays a partial mediating role in the influence of empathy on prosocial behavior, while there is still a significant direct effect. An increasing number of researchers agree that most research results are partial mediations when mediating variables are correctly manipulated and tested, because partial mediations do not mean that data results are not perfect; it may mean that there is not only one mediation path for independent variables to influence dependent variables. Other mediating variables are worth exploring in the future (49, 50). This suggests that in addition to social responsibility as a partial mediator, other mediating variables, such as solidarity, emotion, gratitude, and social support may exist in the influence path of empathy on prosocial behavior, which requires further consideration.

### Limitations and Future Directions

First, this study examined only the correlation between empathy and prosocial behavior, so we cannot infer a causal link. Future studies can investigate whether a causal relationship exists between empathy and prosocial behavior during a pandemic through a more rigorous experimental design. Second, a longitudinal study design would be more effective to obtain the developmental trend of the relationship between empathy and prosocial behavior in the context of a pandemic. Third, the participants of the study resided in low-risk areas of the pandemic, so the applicability of results to higher-risk areas is limited.

In addition, the results of this study have a positive reference for the psychological construction of college students in the context of the pandemic. The country, society and schools can cultivate college students' empathy and social responsibility in various ways, so as to promote more prosocial behaviors of college students and improve their mental health. To be specific, college mental health education can carry out mental health courses with the theme of cultivating empathy, and college moral education courses can cultivate college students' social responsibility through social responsibility education courses and different kinds of social practice activities.

Future researches can investigate the manifestations of new prosocial behaviors, such as wearing masks and maintaining physical distance, and develop measurement tools suitable for prosocial behaviors in the context of pandemic, so as to better study the influencing mechanism of prosocial behaviors and its relationship with mental health in the context of pandemic.

## Conclusion

This study found that in the context of COVID-19, college students' empathy can positively predict prosocial behavior and social responsibility, and social responsibility can positively predict prosocial behavior, and social responsibility plays a partial mediating role in the impact of empathy on prosocial behavior.

## Data Availability Statement

The raw data supporting the conclusions of this article will be made available by the authors, without undue reservation.

## Ethics Statement

The studies involving human participants were reviewed and approved by Northwest Normal University, Development of Psychology. Written informed consent to participate in this study was provided by the participants' legal guardian/next of kin.

## Author Contributions

YJ and SW designed the study. YJ supervised the project. YJ and XZ helped revise the manuscript. YY analyzed the data and drafted the manuscript. SW collected the data. All authors have read and approved the final manuscript.

## Conflict of Interest

The authors declare that the research was conducted in the absence of any commercial or financial relationships that could be construed as a potential conflict of interest.

## Publisher's Note

All claims expressed in this article are solely those of the authors and do not necessarily represent those of their affiliated organizations, or those of the publisher, the editors and the reviewers. Any product that may be evaluated in this article, or claim that may be made by its manufacturer, is not guaranteed or endorsed by the publisher.
